# Development of an Assay for Soy Isoflavones in Women’s Hair

**DOI:** 10.3390/nu14173619

**Published:** 2022-09-01

**Authors:** Souad Bensaada, Isabelle Raymond, Malena Breton, Isabelle Pellegrin, Jean-François Viallard, Catherine Bennetau-Pelissero

**Affiliations:** 1 Carreire Campus, Sciences and Technology Department, Pharmacy Faculty, University of Bordeaux, 33076 Bordeaux, France; 2 ARNA, U1212 Inserm, 5320 CNRS, Pharmacy Faculty, 33076 Bordeaux, France; 3 CHU Bordeaux, USN B0-Hôpital Haut Lévêque, 33604 Pessac, France; 4 CHU Bordeaux, Laboratory of Immunology and Immunogenetics, Resources Biological Center (CRB), 33000 Bordeaux, France; 5 Bordeaux Sciences Agro, 33175 Gradignan, France

**Keywords:** soy isoflavones, consumer exposure, hair samples, dietary inquiry, digestion method

## Abstract

Soy isoflavones, at adequate dosages, have estrogenic and anti-thyroidal effects in animals and humans, which can either be beneficial or adverse, depending on the consumer’s physiological status. Hence, this study presents an assay of soy isoflavones in hair, aiming to give new information about a person’s exposure to isoflavones, when health issues related to estrogenic or thyroidal effects are observed. Aqueous or organic extraction procedures following acidic, basic, or enzymatic digestions were tested on 60 hair samples (from volunteers) from a hairdresser, and a clinical trial 2017T2-29. The acidic digestion method was the most efficient regarding isoflavones. A specific inquiry was developed to assess the dietary habits of French consumers based on the analysis of 12,707 food labels from France. It was used to check for the reliability of the new assay method. A score for the consumer exposures to isoflavones was built considering, among other parameters, soy-based diets and foodstuff containing soy as an ingredient, i.e., “hidden-soy”. The correlation between this score and isoflavone measurements in hair reached 0.947; *p* < 0.001. Therefore, providing that relevant data are considered to assess isoflavone exposure, hair that smoothens daily isoflavone intake variations, is a relevant tissue to assess human isoflavone exposure for subsequent health analyses.

## 1. Introduction

Soybean has been used as a dietary source in Asia for likely more than 3000 years [1]. Basically, soybean, as a pulse, is rich in protein (over 40% of crude composition), polyunsaturated fatty acids (ω3 and ω6), and fiber. In addition, soy is one of the pulses best suited for animal feeding due to its good amino acid profile. This exceptional composition gives soy one of the best nutritional values among all legumes. However, besides its fabulous composition, raw soy, as other legumes, contains many anti-nutritional factors that reduce its palatability and digestibility [2]. This developed defensive biochemical arsenal consists mainly of phytic acids, tannins, oligosaccharides, haemagglutinins, lipoxygenases, anti-trypsin factors (Bowman–Birk and Kunitz), allergens, and isoflavones with hormonal activities [2]. However, the latter is not the least as they were shown to reduce the herbivore’s fertility in both breading and wild conditions [3,4]. Moreover, some beneficial effects have been reported on human health [5,6].

While traditional Asian cooking recipes were able to remove estrogenic isoflavones from soy, thanks to prolonged cooking and simmering in water, modern industrial preparations no longer involve these water steps [7]. Consequently, the current human exposure to estrogenic isoflavones is probably the highest it has ever reached in human history. In addition, the environment is a source of other endocrine disruptors, including wrapping agents, pesticides, plastic stabilizers, flame retardants, and other anthropogenic substances [8].

This study was performed in the frame of a clinical investigation: ISOLED 2017T2-29, which aims to analyze the potential link between isoflavones exposure and the occurrence of systemic lupus erythematosus (SLE) crises. SLE is an autoimmune disease known to exhibit a highly unbalanced sex ratio occurrence [9]. Ninety percent of the SLE subjects are women and the current hypotheses link this occurrence to genetic factors bearded by the X chromosome and to estrogen impregnation. Indeed, SLE progression is not observed in pre-adolescent girls or post-menopausal women. The role of environmental factors in SLE occurrences is nowadays under debate [10]. Among other environmental factors are estrogens and phytoestrogens from soy. Little is known about this potential association; this may be due to the lack of reliable data on exposure to estrogenic isoflavones.

To correctly determine soy isoflavone’s health effects on humans, it is crucial to assess their exposure in the best possible way. Some studies have tackled this issue by attempting to correlate dietary intakes to isoflavones concentrations in human urine or serum and plasma samples as biomarkers of exposure. In this context, a comparative review [11] highlighted that in many circumstances the correlation was shown to be poor. Consequently, developing an assay on hair samples seemed of interest to improve the knowledge of human exposure to soy isoflavones. The sustaining hypothesis is that hair is a better candidate as it smooths daily isoflavones intake variations as well as the variations of biological fluid concentrations. Therefore, even though no one has attempted (until now) to assay soy isoflavones in hair or use this measurement as a biomarker of exposure, both issues are described here. Such knowledge may open up new perspectives in analyzing the health effects of isoflavones. However, hair is a very specific matrix since it presents great resistance to biochemical, chemical, and physical treatments. Therefore, the challenge is to achieve efficient hair digestion to recover the largest portion of substances of interest without altering them.

In this paper, several methods were used to digest hair samples and extract isoflavones and are presented alongside their respective isoflavones recovery rates. A dietary inquiry was set up to assess chronic isoflavones exposure and dietary hair scores were derived from it, taking into consideration some hair parameters. A Pearson correlation coefficient was obtained between these scores and isoflavones measurements in hair and compared to the data reported in the literature.

## 2. Materials and Methods

### 2.1. Materials

Hair samples were collected in two locations. The first set (of 8 samples) was anonymously collected from female volunteers aged 35 to 85 at a hairdresser (with full consent from volunteers). These women accepted answering the food habit inquiry. The second set (of 52 hair samples) was anonymously collected from premenopausal women with or without autoimmune diseases, including SLE, at the hospital, in the frame of the 2017T2-29 clinical trial (ISOLED study). In this trial, in addition to hair—blood, and urine samples were collected in healthy premenopausal women and patients with SLE or other autoimmune diseases. The recruitment is ongoing, with the objective of reaching a minimum of 72 volunteers: 33% healthy, 33% SLE patients, and 33% patients with other autoimmune diseases, with a less unbalanced sex ratio. The only other autoimmune disease currently diagnosed among the volunteers is immune thrombocytopenic purpura. The aim of the clinical study is to examine a potential difference in isoflavones exposure between SLE patients and the other volunteers, by assessing isoflavones in all biological samples. Urine and plasma samples were not used in this study as it focuses on the new IFs assay in hair samples. All volunteers agreed to answer the dietary habit inquiry designed for this study and signed a full informed consent. The volunteers were on their casual diets and no selection was made based on soy consumption habits. Hair sampling was standardized as in most scientific works. It was performed at the back of the skull, at the nape of the neck [12]. 

#### 2.1.1. Direct Extraction Tests

Hair samples were crushed on a Minilys^®^ apparatus from Bertin Technologies (France) for 4 cycles of 1 min each at full power. For this operation, samples were placed in Precellys^®^ Lysing kits (P000917-LYSK0-A). Isoflavones were extracted directly using either methanol, ethanol, or ethyl-acetate. All solvents were from Sigma-Aldrich™.

#### 2.1.2. Digestion Tests

Hair digestion was implemented by chemical or biochemical reactions on two types of samples. Tests were performed on 50 mg hair either pre-cut or pre-cut then crushed (Minilys^®^). Unless otherwise mentioned, all reagents were from Sigma-Aldrich™. The chemical digestion was carried out using either NaOH 1 N or HCl 0.1 N. The reagents were diluted in ultrapure water from an Elga Veolia^®^ water instrument. The biochemical digestion was tested using Pronase (SIGMA, Sigma Aldrich Chimie S.a.r.l, France, P5147) or Keratinase (SIGMA, K4519) solutions. Both enzymes were dissolved in a digestion buffer, which was Tris buffer pH 8.25 0.05 M with 100 units penicillin G/mL (SIGMA, P-3032), 1 mM streptomycin, and 1 mM EDTA. Stock solutions were kept frozen (−22 °C) in black storing vials. The Keratinase stock solution was at a concentration of 180 Units/mL in water. The Pronase stock solution was at a concentration of 20 mg/mL in a phosphate buffer pH 7.4 0.01 M with (CH_3_CO_2_)Ca 10 nM. For each digestion test, 2 mL of digestion solution was used.

#### 2.1.3. Digestion and Extraction of the Conjugated Isoflavones

The digestion of isoflavone conjugates (glucuronides and sulfates) was performed with β-glucuronidase-aryl sulfatase from *Helix pomatia* (Roche, 10127698001) diluted (5 µL/mL) in sodium acetate buffer 0.1 M, 0.14 M EDTA, 100 UI/mL penicillin G (SIGMA, P-3032), and 0.1 mg/mL streptomycin (SIGMA, S-6501) pH 5. The extraction of aglycone compounds was then performed using acidified ethyl-acetate (500 µL HCl 38% per L).

#### 2.1.4. ELISA Measurements

All salt reagents were from VWR-France. The protocol also involved thyroglobulin from swine (SIGMA, T1126), bovine serum albumin (EUROMEDEX, 04-100-812-C), and secondary antibody goat anti-rabbit IgG antibody-Amdex (SIGMA, RPN4301). The revelation steps required o-phenylenediamine dihydrochloride –OPD– (SIGMA, P1526). Stock solutions were prepared at 10 mg/mL in water and stored in black vials at −22 °C. Glassware in contact with unconjugated isoflavones was coated with Sigmacote^®^ (SIGMA, SL2). The sample, washing, and assay buffers were phosphate buffer saline 0.01 M, 0.9% NaCl, 0.2% Tween, 1% DMSO, and pH 7.3. To obtain the saturation and antibody buffers, 1.6 mg/L of bovine serum albumin was added to the latter. The revelation buffer was citrate-phosphate buffer 0.15 M, pH 5 with 0.05 mg OPD/mL. The stop solution was H_2_SO_4_ 4 M. The primary antibodies obtained on various functionalized isoflavones were selected based on their specificity and the sensitivity obtained by applying the ELISA procedure [13,14,15]. For each hapten initially synthesized, 2 rabbits were injected and the best polyclonal antibody was retained for further use in the assay. These antibodies were harvested if required to avoid undesired cross-reactions with the unwanted antigen.

#### 2.1.5. Control Solutions for Digestion Tests

Several pure compounds were used as control solutions to assess the digestion and extraction recovery. The first was Genistin from EXTRASYNTHESE™ (Ref. 1325 S) and was dissolved at 1 mg/mL in DMSO as the stock solution. Then, genistein-O-7-sulfate, Ref. G350045, and genistein-7-b-D-O-glucuronide, Ref. G350015, were from TRC Canada. Their stock solutions were dissolved at a concentration of 2 mM in DMSO and were stored at −22 °C.

### 2.2. Methods

#### 2.2.1. Dietary Inquiry

The dietary inquiry used in this study is given in the Supplementary Material S1. It was drawn out to focus on potential isoflavones exposure. It was adapted to the French food market offer and the French diet habits. From a previous study, in which more than 12,700 foodstuff labels were analyzed [16], it was possible to determine in which foodstuff and transformed dishes soy could be incorporated. The occurrence of soy as an ingredient, i.e., “hidden-soy”, was known to be more frequent in collective restaurants than in foodstuff bought in supermarkets [16]. Therefore, the inquiry addressed the usual location of food intake. It also recorded the following items:

- The dietary specificity, such as vegetarian, Asian, or gluten-free diets;
- The eater’s status, i.e., as big, medium, or small;
- The snacking practice;
- The main cooking practices;
- The intake frequency of soy-based foodstuffs but also of food that contain “hidden-soy”.

An estimation of the food portion size was also collected, although a retrospective estimation could never be of high reliability. However, such data could validate the eater status given elsewhere. As the inquiry was used to validate the hair measurements, some parameters were also documented in line with the specificity of these samples, such as the frequency of haircuts and shampooing. Finally, the duration of a given described diet was related to the hair length to determine whether the hair sample has always been exposed to the same diet (see Supplementary Material S1 for details).

#### 2.2.2. Isoflavones Food and Hair Scores

A food score was attributed to foodstuff considering the average isoflavones content [17] and intake frequency by the volunteers (see Supplementary Material S2). This was based on the known isoflavones content of 240 French foodstuffs. The average content combined both mean isoflavones level measured in a food category and the occurrence of food containing soy in this given category. In Lee et al. [17], the concentration values of genistein (GEN) and daidzein (DAI) expressed in mg/100 g of food or mg/portions were obtained using ELISA and, therefore, were expressed in aglycone equivalents. The calculation of both the Isoflavones Food Score (IFS) and Isoflavones Hair Score (IHS) can be found in Supplementary Material S2. Basically, the IFS was composed of the sum of the food scores multiplied by a factor considering, on the one hand, the intake quantity, and on the other hand, some hair parameters, such as cutting and treatments, e.g., shampoo, coloring, perms, and their frequencies, which are shown to marginally influence the xenobiotics measurements. Meanwhile, the IHS was adjusted from the IFS using a potential dilution factor considering the hair length and diet duration. See Supplementary Material S2 for details on the calculation.

#### 2.2.3. Sample Preparation 

Hair samples collected either at the hairdresser or at the hospital and duly identified, were stored in plastic vials at −22 °C until processing, at the Bordeaux Hospital biobank CRB (Bordeaux Biothèques Santé Centre de Ressources Biologiques, Bordeaux University Hospital). 

Hair samples were first cut into small pieces of less than 1 mm long. Preliminary assay tests were performed directly on 50 mg of such samples (soda, acid, pronase, and keratinase digestions). However, this cutting was found insufficient and was then associated with Minilys^®^ crushing for which 50 mg of hair was distributed into 3 different Precellis^®^ tubes containing 6 metal beads. Crushing was performed on a 4-cycle basis, each cycle lasting 1 min. The crushed samples were transferred into 40 mL screwed top glass vials, coated with silicone, in which the digestion was performed at the relevant temperature and duration in 2 mL of the corresponding solution (see Table 1 for procedure summary). The samples were then brought to 100 °C for 10 min and cooled to room temperature to deactivate the proteases if the latter were used for the digestion. 

After pH neutralization, and when relevant (see Table 2), 2 mL of 0.1 M of acetate buffer was added to the vials with 10 µL of b-glucuronidase aryl-sulfatase solution. The samples were then incubated overnight at 37 °C under 200 rpm shaking to release the aglycone compounds [18]. The latter were extracted directly in the glass vials using 3 mL of acidified ethyl-acetate. Namely, they were extracted 3 times and evaporated to dryness using a Speed-Vac (Thermo-Electron^TM^ Corporation, Fisher Scientific, Illkirch, France). At each step, the vials were vortexed for 30 seconds, centrifuged at 500 g (Jouan^TM^ CR3, Fisher Scientific, Illkirch, France) for 10 min at 4 °C, and stored for 1.5 h at −22 °C to allow phase separation. The aqueous phase froze while the ethyl-acetate phase which contained the isoflavones in aglycone forms remained liquid. The three ethyl-acetate phases of the same sample were collected and evaporated to dryness in the same hemolyzed tube precoated with Sigmacoat^®^. The conjugate hydrolysis—either by b-glucuronidase aryl-sulfatase or acid digestion—was monitored using genistein-7-sulfate as a control reagent in an external standard run in parallel to the samples. This hydrolysis performance was always between 97 and 101%. After final evaporation, samples were diluted in 1 mL of assay buffer and when required sonicated using an ultrasound probe (LCD Series Ultrasonic Processor). Samples were stored at −22 °C until assay processing.

#### 2.2.4. ELISA Measurements in Hair

GEN, DAI, and equol were assayed in the hair samples using specific ELISAs as described previously [15]. Haptens functionalized on different carbon atoms were previously synthesized [13,14]. Specific polyclonal antibodies were obtained from rabbits, for each isoflavone bound to bovine serum albumin and the best antibodies were retained for assay development. Standard curve preparations and sample dilutions were performed in silicone-coated glass vials. The ELISAs followed a competitive procedure with an immobilized competitor which was the homologous hapten bound to swine thyroglobulin. The sensitivities varied between 0.08 ng/well and 0.4 ng/well; intra-assay variation was always below 7% and inter-assay variation was below 17%. For the hair samples, final dilutions varied between 1:5 and 1:20. Each hair sample was assayed in triplicate using three extracts and on three different microtitration plates. All values are given in aglycone equivalents.

#### 2.2.5. Statistical Treatments

Each concentration value was always a mean and standard deviation of at least triplicate measurements except when repeatability was assessed. In that case, the number of replicates was given. Due to the large cohort analyzed, data followed a normal distribution. Therefore, the correlation between IFS or IHS and isoflavones measurements as well as their significance were calculated following the Pearson procedure. The significance was obtained by comparing the ν value with data from Student significance tests.

## 3. Results

### 3.1. Tests of the Extraction Methods for Isoflavones from Hair Samples

#### 3.1.1. Tests on Pure Control Substances 

The results of several extraction and digestion tests performed on pooled hair samples are reported in Table 2. The higher recovery rate for genistein-7-sulfate was recorded with the acid digestion using HCl 0.1 M for 8 h at 40 °C. However, this digestion was not able to recover the genistein-7-β-D-glucuronide metabolite. This could indicate that HCl most probably hydrolyzed the isoflavone moiety and destroyed it. Moreover, it can be observed that soft digestion processes using pronase or keratinase were able to preserve the conjugates, i.e., both genistein-7-sulfate and genistein-7-β-D-glucuronide, although the best overall recovery was obtained using keratinase. Direct methanol and ethanol extractions were not very efficient compared to acid treatment. Finally, the use of genistein as recovery control led to singular results. Indeed, NaOH treatment, leading to hair sample dissolution, was more efficient on hair digestion than any other treatment tested, and this includes the enzymatic and acid treatments. However, it did not significantly retrieve isoflavones sulfo- and glucurono-conjugates from pure solutions or hair samples (see Table 2).

#### 3.1.2. Test on Hair Samples 

Interestingly, although the acid treatment was not able to retrieve the glucuronide conjugates, it was the treatment that led to the highest dosages whatever the hair samples considered (see Table 2). Next to the acid treatment, NaOH treatment gave highly variable values not significantly different from 0. Pronase and keratinase digestions led to significant isoflavones assays although lower than those determined using the acid treatment, except for pronase on the low-concentrated hair sample (see Table 2). The latter was a mixture of several samples collected on soy non-eaters. The high-concentrated hair sample was a mixture of several samples collected on soy-eaters. The mixture sample was a pool of three samples: one came from a high soy-eater, the second from a medium soy-eater, and the third from a non-soy-eater. Crushing using Minilys^TM^ reduced the inter-assay variations (see Table 2). This could be seen on all treated samples. Likewise, the inter-sample variation is higher for the low-concentrated sample compared to the high-concentrated one, independently of the digestion treatment used. These low-concentrated samples were close to the quantification limit.

### 3.2. Characterization of the Isoflavones Assay in Hair

#### 3.2.1. Sensitivity

The specificity of the primary antibodies allowed to assay hair samples at dilutions as low as 1:5 without a pre-washing procedure. Avoiding pre-washing steps allowed to minimize losses of desirable molecules. The sensitivity of GEN assay was based on the IC_50_—half maximal inhibitory concentration of the standard—curve, i.e., 20 ng/mL. Then, since assay samples were 50 mg each, the sensitivity was 2 ng/mg of hair for GEN. However, the quantification and the detection limits for GEN were 0.8 ng/mg and 0.5 ng/mg of the hair samples, respectively. Moreover, the sensitivity of DAI assay, based on the IC_50_ of the standard curve was 13 ng/mL. Then, the sensitivity was 1.3 ng/mg for DAI. However, the quantification and the detection limits for DAI were 0.41 ng/mg and 0.12 ng/mg of the hair samples, respectively. Finally, the assay sensitivity for equol, based on the IC_50_ of the standard curve was 6.8 ng/mL. Then, its sensitivity was 0.7 ng/mg. However, the assay and the detection limits for equol were 0.1 ng/mg and 0.08 ng/mg of the hair samples, respectively. 

#### 3.2.2. Specificity

As for other immunological tests, the specificity relies on the antibodies used. In ELISA tests several dilutions of the same sample are currently done in order to obtain values as close as possible to the IC_50_ of the standard curve. If non-specific bindings had occurred, it would have been noticed since the different dilutions would give rise to highly different concentrations. Here, most of the samples had to be diluted at 1:5 and 1:10, increasing, by the way, the potential occurrence of cross-reactions with undesired compounds. However, there were good similarities between the concentrations results obtained at those different dilutions. This indicates that undesired cross-reactions were very limited.

#### 3.2.3. Repeatability and Reproducibility

Tests were performed repeatedly on different samples assayed on different microtitration plates. Therefore, the results given in Table 3 are inter-assay variation coefficients. As mentioned in this table, data were from the same extraction for samples 26 and 28 while they were from 2 different extractions for the soy-eaters hair sample. The coefficient obtained on the same extraction, expressed the repeatability, while the data obtained from two different extractions gave the reproducibility. 

In all cases, the inter-assay variation coefficients were always below 25% and seemed acceptable. However, it should be mentioned that nearly 35% of the 60 samples assayed were close to the quantification limit and 18% were at the detection limit.

### 3.3. Correlation with the Dietary Data

**Figure 1:**
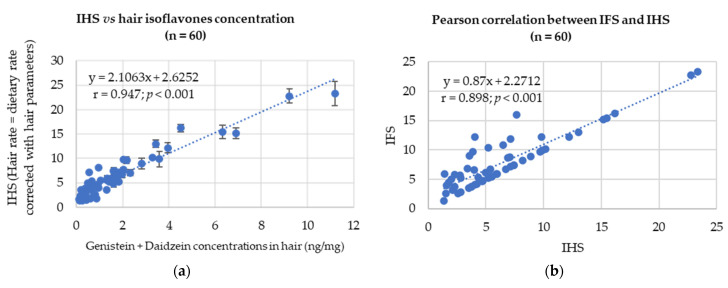
Pearson correlations between HIS and either total hair isoflavones or IFS, calculated for the whole 60 volunteers (*p* < 0.001): (**a**) Pearson correlation between IHS and hair isoflavones (GEN+DAI) concentration; (**b**) Pearson correlation between IFS and HIS.

In order to validate this new extraction and assay, it was decided to check its reliability based on specific dietary habit inquiries. The results are shown in Figure 1.

As can be seen in Figure 1a. A large amplitude was observed in isoflavones measurements with the minimum level of GEN + DAI being 0.11 ng/mg and the maximum being 11.18 ng/mg. This reflected the IHS which varied from 1.39–23.32. Figure 1b gives the correlation between the IFS and the IHS. The Pearson regression coefficient r between IHS and isoflavones levels in hair was 0.947 based on 60 sets of data (*p* < 0.001). Its significance was good with less than 1 chance over 1000 to have no correlation. The r coefficient was also 0.914 for GEN alone with *p* < 0.001 and 0.91 for DAI alone with *p* < 0.001 for Pearson correlations (see supplementary Material S4). Moreover, the Pearson correlation between IFS and IHS was also significant and high when based on 60 pairs of values with an r value of 0.898 (*p* < 0.001).

### 3.4. Detection of Equol

Equol was assayed for the first time in 55 of the hair samples and detected in 34 of them. This represented 61.82% of the population. However, levels were consistently low and for 67% of the equol positive samples, the concentration value was close to the detection limit.

## 4. Discussion

Isoflavone contents were measured in human hair samples and applied to the assessment of isoflavones exposure for the first time. So far, urine, serum, and plasma samples have been the only biological materials used to detect isoflavones as biomarkers of exposure [11]. 

### 4.1. Reliability of Isoflavones Assessment in Hair Compared to Other Biological Samples

Table 4 provides an overview of different studies, using either plasma or urine biomarkers, allowing a comparison with the present work. As can be seen, the correlation coefficient obtained between soy intake and measurements of GEN + DAI is one of the highest among those reported so far in the literature. 

#### 4.1.1. Comparison with Urine Samples

Urine, as it basically concentrates the blood substances, can be used to explore low isoflavones intakes. Compared to isoflavone concentrations in hair, urine-associated levels can be 100 times higher. However, the measurement can only reflect an exposure that occurred within the last 48 h. Indeed, in some of the studies reported in Table 4, as urine was collected on a 24- or 48-h period, a highly reliable exposure evaluation was obtained. However, such a sampling may not always be possible in large cohorts. Other than that, the r values, correlating urine isoflavones measurements and dietary intake recording soy, are as low as 0.27 [25]. Indeed, spot urine samples have given less accurate results compared to 24- or 48-h urine collection but can be used with a certain reliability provided that they were adjusted on urine creatinine levels. On another side, the highest r value (0.97), obtained from a Spearman calculation, in fact corresponds to a small number of subjects (14) for whom it was hence possible to estimate the isoflavones intake and biomarker intensity with high accuracy. When the number of subjects is larger than 50, the Pearson correlation coefficients obtained range between 0.27 and 0.52. This could be explained first by the possible large isoflavone–daily-intake variations and second by the fact that isoflavones concentrations in urine depend greatly on the timespan after ingestion. As pointed out by Shinkaruk et al. [15], after an isoflavone challenge, the urine C_max_—maximum concentration in body fluid after ingestion—was observed between 8 and 16 h, depending on the isoflavone type measured. However, a daily intake could also lead to pharmacokinetic steady-state levels [26]. However, apart from experimental situations, this latter case has been only observed with food-supplement intakes or for chronic heavy-soy-consumers. Hence, depending on the urine sampling time, the measurements could yield very different results, even for identical isoflavone exposures. Consequently, if timespan between intake and sample collection is not recorded, then the correlation based on unadjusted data could be falsely low. Finally, urine samples, such as hair samples, are usually obtained in a non-invasive way. This aspect constitutes an advantage over blood samples.

#### 4.1.2. Comparison with Plasma or Serum Samples

In the literature (see Table 4), when plasma or serum samples are measured, they usually correspond to spot sampling. Sometimes, the time of sampling is standardized, for instance between 7:00 am and 8:00 am, after overnight fasting. Some other times, it is arbitrary and even not mentioned, although, as already referred to, the pharmacokinetic of soy isoflavones orally administrated is well documented [15]. For GEN, the T_max_—time at a maximum concentration in body fluid after ingestion—observed on 18 male volunteers, was shown to occur 7.33 h ± 2.38 h after ingestion with an elimination half-life T_1/2_—time required for the elimination of half concentration of a given substance from plasma—of 22.2 h ± 13.38 h due to individual recirculation phenomena. For DAI, the T_max_ was shown to occur 8.78 h ± 2.84 h after ingestion with an elimination half-life T_1/2_ of 11.2 h ± 4.51 h. Consequently, if the time of sampling is not standardized, high variability in isoflavone measurements is likely to happen even for identical food intakes, resulting in weak correlations. It is thus not surprising that the published correlation coefficients between soy-food intake and serum measurements are as low as 0.27 [30]. Concerning the highest r values observed for plasma correlation, it is possible to point out some objective reasons for these situations. For instance, the value 0.92 [32] leans on the particularity of the study as even though performed on only 14 volunteers, this small cohort size allowed an accurate isoflavones intake assessment made by weighted food diaries. The other study reporting a high r value (0.80) for plasma correlations is proposed by Verkasalo et al. [27]. The authors performed a Spearman correlation analysis since the 80 consumer profiles used for their study were distributed into 4 groups only, according to their soy-food intake. They used a statistically unsatisfactory process as only 4 mean isoflavones blood levels—4 values for GEN and 4 values for DAI—were deduced and used as a basis to obtain the correlation, instead of the whole 80 data. In fact, apart from these two interesting yet questionable studies (Ritchie’s and Verkasalo’s), the coefficient r correlating plasma isoflavones levels and dietary records is always below 0.55, as shown in Table 4. Conversely, the daily isoflavones variations in biological fluids were invisible in hair, by the way giving insights about long-term exposure. Finally, if 24- or 48-h dietary surveys could be suitable for isoflavones assessment in biological fluids, a new dietary questionnaire recording global French food habits focusing on soybean intakes had to be built to validate hair measurements.

#### 4.1.3. The Importance of the Food Intake Estimation

Of course, a good correlation between isoflavone measurements in body tissues and food intake relies on the quality of isoflavone measurements and the reliability of the food inquiry. Past food records were not as accurate as newer ones primarily because the isoflavones concentrations in soy ingredients incorporated into processed foods were underestimated. For instance, in 2006, correlations between isoflavones and dietary records or food databases in the USA ranged from 0.41–0.55 [36]. Although these coefficients were in the same range as those published later, the initial database essentially focused on soy-based products, under-considering products containing “hidden-soy”. These could be, for instance, bread in which soy flour was incorporated to whiten the breadcrumbs. In the present study, the food inquiry was mainly focused on soy-based food but also on French foodstuff containing “hidden-soy”. These were primarily elaborated dishes or foodstuff based on meat or fish that were previously crushed and restructured. In France, it was shown [16] that soy was not used to whiten breadcrumbs, but broad bean meals could be used in industrial soft bread. Contrary to soybean, broad-bean flour contains only traces of GEN and DAI. Thus, soft bread was not retained in this inquiry. More globally, the recorded items choice was based on the study by Lee et al. [16]. This previous analysis of the French foodstuffs’ labels determined which could possibly contain soy as an ingredient. The comprehensive analysis of 12,707 food labels included transformed foodstuff containing minced ingredients [16]. The food categories that were investigated included a large panel of products. For instance, 2 dairy products out of the 2146 analyzed were shown to contain soy. So, dairy products were not assessed in the present inquiry. Moreover, 65 out of 154 labels of minced beef–meat were found to contain soy and, therefore, frozen minced beef portions were analyzed in the present study. Hence, as can be seen in Supplementary Materials S1 and S2, the inquiry recorded: soy juice, soy yogurt, soy cream and ice cream, soy cheese and tofu, other plant-based juices, other soy products (miso, tempeh…), meat-based dishes, fish-based dishes, vegetables based dishes, pizzas, elaborated dishes, canned recipes, nuggets, hamburgers, kebabs, meatballs, escalope cordon bleu, stuffed vegetables (tomatoes, cabbages), frozen minced steaks, industrial biscuits, other legumes, chickpeas, soy seeds, soybean sprouts, alfalfa or clover sprouts. In the score-determination-tool, the portion size was only addressed through a question dealing with the eater status, i.e., big–eater, medium–eater, or small–eater. Questions were addressed to refine the consumption habits. Some dealt with the consumption mode, i.e., vegan, vegetarian, lacto-vegetarian, flexitarian or omnivorous. Others addressed the type of the main meals, i.e., Asian, gluten-free, lactose-free or other. Indeed, it was shown [16] that gluten-free French products could more frequently contain soybean than conventional ones. Moreover, some questions assessed the main cooking practices. For instance, wok or oven have slight effects on isoflavones concentrations. Conversely, boiling in water can reduce isoflavones content significantly. Then, the main meal-place—canteens, fast-foods, restaurants, home, …—was recorded, as it was shown [16] that meals taken in canteens or fast-food restaurants could bring more isoflavones than those prepared at home. Finally, the IFS was equal to the sum of all food scores multiplied by a factor considering hair treatments and eater status. The IHS was obtained by multiplying the IFS by a factor including the duration of the described diet and the hair length. The latter represents a dilution factor introduced because the overall hair sample length was analyzed in the assays. The regularly high correlation factors obtained in this study suggest that this dietary approach is correct and takes sufficiently into account the isoflavone intake, including the one provided by “hidden-soy”.

### 4.2. Positive Innovative Elements Brought in This Work

#### 4.2.1. The Assay

Even if 35% of samples were close to the quantification limit in this panel of volunteers, the correlation between isoflavones measurements and the IHS is good (Figure 1). Because soy consumption is still low in France, it was not expected to find high levels of isoflavones in all hair samples. Nevertheless, only 11.6% of the samples assayed were considered as completely free of isoflavones. This reinforces the idea that soy and isoflavones intakes are significant in the French population [17] even if the level of consumption is not always of concern. The assay was also able to detect abnormalities in dietary declarations as will be seen below. Finally, it is also the first time that equol is measured in hair. The proportion of equol producers was found to be 61.82% of 55 volunteers. A similar proportion was found in 2006 in a panel of 60 French women, using the same assay technique [26]. Indeed, it is important to consider the sensitivity of the assay technique while assessing the equol–producer percentages in a population. Hence, in the present study, even if ELISA has a great sensitivity, a large proportion of samples were close to the detection limits and those samples might have gone undetected by other assay methods.

#### 4.2.2. The Food Survey

Here the measurements were validated by the food inquiry which was itself validated by the hair measurements. As can be seen in the Supplementary Materials S1 and S2, the inquiry was targeted to specific foodstuffs containing soy and isoflavones [16,17]. Thus, the inquiry did not take too much time by itself and could be proposed to the volunteers. Moreover, as mentioned earlier, the food intake gave information data, but these had to be adjusted to obtain an IHS which reflected better the isoflavones hair content. Hence, the hair basic color and treatments applied to hair were considered including washing, cutting, coloring as well as perm frequency. The duration of the claimed diet was also very important to consider since, here, the all hair-length were used for measurements. Indeed, due to a mean growth of about 1 cm/month, the last 6 months consumption was only reflected into the 6 cm of hair proximal to the skull. Hence, if the diet was changed for soy, only recently, the trace of the consumption may be diluted if the volunteers wore long hair. Such a phenomenon was initially observed while collecting the first data in this study and led to the corresponding adjustment. 

### 4.3. Challenges of the Present Analytical Method

#### 4.3.1. Digestion Procedure

As a first attempt to assay isoflavones in hair, this work presented some difficulties. The first one is linked to the digestion method retained. NaOH 1 M treatment described in Gaillard et al. [19] was not adequate for isoflavones. This treatment destroyed the isoflavone-conjugates used as controls except for Genistin. Because Genistin is only present in plants and not in human biological fluids, it is not expected to be present in hair. On hair samples it retrieved isoflavones at levels not significantly different from 0. From multiple tests, it clearly appeared that acid digestion using HCl 0.1 N had given the best recovery results on hair samples. However, as it was observed with Genistein-7-β-D-Glucuronide as control, this digestion process destroyed the glucuronide conjugates, including those potentially present in hair. Interestingly, while the enzymatic procedures better preserved these conjugates, they were not able to retrieve more isoflavones from hair samples. Indeed, as mentioned in Table 2, the pronase and keratinase hair digestions were followed by β-glucuronidase-aryl-sulfatase hydrolysis. The latter was performed to hydrolyze the conjugates into aglucones, if any were present in the hair samples. This step, which generated free GEN, DAI and equol allowed a 98% recovery of the external standards via the ethyl-acetate liquid-liquid extraction. Therefore, it was expected that the enzymatic method preserving the glucuronide conjugates would lead to higher isoflavones values in hair samples. However, it was not the case, when comparing the recovery percentages obtained on hair samples with keratinase or acid digestion. It was assumed that the keratinase treatment was not able to digest the hair matrix as much as the acid treatment and therefore that the isoflavones and metabolites could not be available for β-glucuronidase digestion. Moreover, pronase gave a better recovery percentage on genistein-7-β-D-glucuronide than acid (61.59% ± 7.85% vs. 00.00%) but a lower genistein-7-sulfate recovery (50.97% ± 0.56% vs. 98.32% ± 16.26%). However, on hair samples, the isoflavone recovery was not better than the one obtained with the acidic treatment. Additionally, it was not retained because the inter-sample variation was higher, especially when applied on concentrated hair samples. The overall recovery observed by enzymatic digestion was never significantly different from the acid digestion. In addition to that, the digestion with NaOH 1 N is not adapted to isoflavones as the recovery rates of both conjugates were null. On the one hand, the hair digestion may not have been efficient enough to release all isoflavones and metabolites. On the other hand, if pronase digestion—compared to the acid one—did not lead to higher values in hair samples, it may be because genistein–glucuronide conjugates were not present. Indeed, looking at the literature, it was observed that the only glucuronide form having ever been found in the hair so far, is ethyl-glucuronide used to assess ethanol addiction [37]. Ethanol being a small molecule, may follow a different process to enter the hair compared to isoflavones. The latter are physico-chemically much closer to steroids. Then, looking at steroid measurements in hair samples, many relevant data were found in the literature. They dealt with cortisol, DHEA, testosterone, androgens and even estrogens. These steroid hormones were mainly detected in hair as either free or sulfate-conjugates [38]. According to Pragst and Balikova [37], the basic and lipophilic character of a substance is important for its incorporation into hair. However, Genistein-7-β-D-glucuronide is a rather strong acid substance with a pKA of 2.74. Meanwhile, Genistein-7-O-sulfate is a strong acid substance with an acid pKA of −2.6. Additionally, free GEN is essentially under a neutral form at physiological pH with a pKA of 7.31 [39]. This property together with the lipophilic nature of GEN may help its incorporation into hair-follicle cells. However, free GEN is far from being the major compound in plasma [40] and if there was no conjugate hydrolysis in hair cells’ vicinity, this would explain why the ELISAs’ sensitivity is challenged while assaying hair samples. DAI and daidzein conjugates follow the same patterns of physicochemical properties. Daidzein-7-β-D-glucuronide pKA is 2.88 and Daidzein-4′-sulfate pKA is -2.4 indicating that both compounds are acidic and strongly acidic respectively in a neutral environment. Meanwhile, free DAI in plasma is lipophilic with poor water solubility and has a pKA of 7.51. Therefore, DAI incorporation in hair can follow the same process as GEN even though the GEN to DAI ratio between plasma and hair may not be completely conserved. Finally, when equol is considered its pKA is 9.63 showing that it acts as a basic compound. It is also lipophilic when not conjugated and these physicochemical characteristics may help its passage into the hair. Conversely, equol-4′-sulfate and equol-7-O-glucuronide have pKA of −2 and 3.27 showing that they are strongly acidic and mildly acidic respectively. Both of them being soluble in water, they are not likely to be incorporated passively in hair bulbs and, therefore, in hair. Nevertheless, the isoflavones concentrations obtained here i.e., between 0.11 and 11 ng/mg of hair, are in the range of other drug levels in hair [37]. 

#### 4.3.2. Limit Linked to the Assay Procedure

The ELISA procedure does not allow the use of internal standards since it relies on specific antibodies. The recovery is always assessed by parallel external standards runs. Moreover, the ELISAs used in this study were based on the recognition of isoflavones aglucones by specific antibodies. The latter could not be used to assay the isoflavone-conjugates directly and required hydrolysis prior to measurements. Thus, the presence of the isoflavones conjugates in hair could still be addressed, by other methods. Such a question is important, since glucuronide–conjugates represent by far the highest proportion of isoflavones forms in plasma or serum [40]. If effectively glucuronides are not able to enter the hair, this could explain the low level of isoflavones found in this matrix. Additionally, as mentioned previously, a large proportion of the measurements of hair isoflavones were close to the quantification limit. Therefore, this technique may be relevant to identify frequent soy consumers but may not be able to distinguish low and very low soy consumers. Nevertheless, only frequent and high soy consumption, exposing people to high isoflavone intakes, have so far been involved in health impairments [41,42]. Therefore, despite these limitations the hair measurements described here may be of help in determining the consumers’ exposure to isoflavones and its health consequences.

#### 4.3.3. Limit Linked to the Dietary Inquiry

Similar to all other dietary surveys, the one developed here for monitoring isoflavones intake relied on the consumer’s declaration. To avoid misinterpretations, several questions were linked to assess the accuracy of nutrition claims and the meals’ locations were recorded. Then, when it was reported that meals were essentially taken outdoors, and if the place was not a gastronomic restaurant, the score attributed to food intake was slightly increased. Despite this, when testing the dietary inquiry at the hairdresser, a large discrepancy was found between the IHS and the hair isoflavones concentration of one of the volunteers who showed some resistance to the study. She was in fact opposed to isoflavones potential negative effects since she belongs to a traditionally soy–eating family. Even though she claimed to have her meals at home, she also claimed to be a non-soy-eater. The Pearson regression r value jumped from 0.633 to 0.989 when her data were not included in the calculation, see Supplementary Material S3 for details. This finding confirms the notion that an answer can be psychologically influenced by the initial question. In our case, the volunteers were always informed about the reasons behind the inquiry, and this may have influenced their answers [43,44]. Finally, as can be seen in Supplementary Material S2, a score is attributed to each foodstuff recorded, depending on the isoflavones level measured, but also on the frequency of soy incorporation if the foodstuff is ultra-processed. For instance, not all nuggets found in supermarkets were shown to contain soy as an ingredient [16] but all contained it in canteens. Thus, the score is only valid in the French population based on recent labels’ analyses. Finally, although this inquiry was applied to 60 people so far, some foodstuffs with high isoflavones concentrations—such as clover sprouts—were never mentioned by any volunteer. Hence, the scores attributed to them are not completely validated.

## 5. Conclusions

The present study reports, for the first-time, measurements of isoflavones in hair and shows that they could be well correlated with dietary records especially built for French consumers. Thanks to acid digestion, the isoflavones–sulfate could be preserved during hair extraction but not glucuronide–conjugates, leaving the question of the presence of conjugated forms of isoflavones in hair without a firm answer. However, this should not prevent the use of hair as a provider of isoflavones exposure biomarkers. This is partly because the use of hair samples to assess a significant exposure of dietary soy isoflavones has proven possible and partly because hair has a remarkable advantage over biological fluids: it can be used to track dietary habits on a long-term basis and, thus, give a more accurate exposure evaluation. Therefore, isoflavone assays in hair samples may be used to monitor certain patient populations at risk of excessive exposure to soy–isoflavones, in particular for pathologies related to estrogens or thyroid hormones. As a result, appropriate dietary advice could be provided to avoid health impairments linked to the risks of interactions between high exposure to isoflavones and pathologies or drug treatments.

## Figures and Tables

**Table 1:** Test of digestions, procedure details.

Digestion Reagent	Solution	Incubation Time	Incubation Temperature
Soda digestion	2 mL NaOH 1 N	1 h	100 °C
Acid digestion	2 mL HCl 0.1 N	8 h	40 °C
Pronase digestion	2 mL 1 mg/mL Tris buffer pH 8.25	24 h	40 °C
Keratinase digestion	2 mL 1.8 UI/mL Tris buffer pH 8.25	2 h	40 °C

**Table 2:** Comparison of the GEN + DAI recovery following different digestion and extraction methods.

Test Samples	Soda Digestion	Pronase Digestion	Keratinase Digestion	Methanol Extraction	Ethanol Extraction	Acid Digestion
Genistein-7-sulfate	0.00% ± 0.00% ***	50.97% ± 0.56% ***	90.39% ± 3.56% ***	54.00% ± 15.00% ***	83.00% ± 8.6%	98.32% ± 16.26%
Genistein-7-β-D-glucuronide	0.00% ± 0.00% ***	61.59% ± 7.85% ***	47.26% ± 6.20% ***	26.00% ± 3.12% ***	8.02% ± 7.56% ***	0.0% ± 0.00%
Genistein	94.00% ± 13.00% ***	96.00% ± 15.00%	97.00% ± 12.00%			56.62% ± 12.12%
Additional treatment	β-glucuronidase digestion					
Positive hair sample	15.82% ± 18.41% ***	74.06% ± 38.62%	55.04% ± 59.37% **			100% ± 10.09%
Negative hair sample	5.86% ± 4.26% ***	106.0% ± 38.00%	24.00% ± 54% ***			100% ± 42.00%
Minilys^TM^ crushing						
Mixture of hair	0.00% ± 0.00% ***	56.42% ± 2.63% ***	64.74% ± 14.07 **	41.34% ± 7.26% ***	18.72% ± 3.08% ***	100% ± 24.86%
References	[19]	[20]	[21]	[22]	[23]	[24]

Significance is given compared to the acid treatment. * *p* < 0.5%; ** *p* < 0.1%; *** *p* < 0.05%.

**Table 3:** Repeatability and reliability tests of the GEN and DAI ELISAs.

		Inter-Assay Variation Coefficients				
		Same Extraction		Different Extractions		
				1st Extraction	2nd Extraction	Overall
GEN	Sample	Sample 28 (n = 6)	Sample 29 (n = 8)	Soy-eater-1 (n = 6)	Soy-eater-2 (n = 6)	Soy-eaters (n = 12)
	Mean ± SD	2.25 ± 0.46	1.18 ± 0.17	3.47 ± 0.35	4.88 ± 0.92	4.18 ± 0.76
	Variation coefficient	20.44%	14.59%	10.12%	18.85%	18.25%
DAI	Sample	Sample 28 (n = 6)	Sample 29 (n = 7)	Soy-eater-1 (n = 6)	Soy-eater-2 (n = 6)	Soy-eaters (n = 12)
	Mean ± SD	1.67 ± 0.28	2.27 ± 0.35	7.86 ± 0.38	6.30 ± 1.53	7.08 ± 1.29
	Variation coefficient	16.63%	15.52%	4.89%	24.34%	18.18%

GEN: genistein in aglucone form; DAI: daidzein in aglucone form.

**Table 4:** Overview of different studies on blood or urine isoflavones as biomarkers of dietary exposures and present work.

Subjects	Nature of Samples	Biomarkers	Dietary Data (mg/day)	Correlation	References
80 British volunteers	Plasma	GEN	7 days food diaries	GEN: r = 0.80; *p* < 0.001	[27]
		DAI		DAI: r = 0.78; *p* < 0.001	
360 women	2 × overnight urine (48 h apart)	ISO	2 days 24 h recall DAI (µg): 5.0–6.4 GEN (µg): 7.3–9.3	ISO: r = 0.52; *p* = 0.001 FFQ: r = 0.29; *p* < 0.01	[28]
77 volunteers	Plasma	GEN	FFQ	GEN: r = 0,53; *p* < 0.001	[29]
		DAI		DIAD: r = 0,45; *p* < 0.001	
284 volunteers 333 volunteers	Urine Serum	ISO	7 days food diaries	Urine r = 0.27; *p* < 0.001	[25]
				Serum r = 0.31; *p* < 0.001	
203 male volunteers	Serum	ISO	FFQ	ISO: r = 0.27; *p* < 0.001	[30]
256 premenopausal women	12 h urine	ISO	FFQ Low: 0.1–2.3 High: 49.8–74.6	ISO: r = 0.51; *p* < 0.001	[31]
14 adults (14% men)	24 h urine Plasma	ISO	24 h food record: 11.0 24 h recall: 12.3	Urine: r = 0.97; *p* < 0.001	[32]
				Plasma: r = 0.92; *p* < 0.001	
51 Japanese women 18 Caucasian women	24-h urine	GEN	48-h dietary recall	GEN: r = 0.54; *p* < 0.001 DAI: r = 0.58; *p* < 0.001	[33]
		DAI			
24 pubertal girls	12 h urine	ISO	3 days 24 h recall ISO: 3.0–13.3	lSO: r = 0.72; *p* < 0.001	[34]
100 healthy women	12 h urine	ISO	24 h recall	ISO: r = 0.460; *p* < 0.001	[35]
60 French women	Hair	ISO	French dietary habit questionnaire	ISO: r = 0.947; *p* < 0.001 GEN: r = 0.914; *p* < 0.001 DAI: r = 0.911; *p* < 0.001	Present study
		GEN			
		DAI			

ISO: isoflavones; GEN: genistein; DAI: daidzein.

## Data Availability

The data presented in this study are available in Supplementary Materials and raw data can be sent upon request.

## Supplementary Materials

The following are available online at https://www.mdpi.com/article/10.3390/nu14173619/s1, Supplementary Material S1: Dietary inquiry proposed to volunteers, Supplementary Material S2: Matrix used to calculate IFS and HIS, Supplementary Material S3: Evidencing a questionable answer to the food questionnaire, Supplementary Material S4: Correlation regression graphs between IHS and GEN or DAI.
