# Supplementary material for: Development of an Assay for Soy Isoflavones in Women’s Hair
Source: Nutrients. 2022 Sep 1;14(17):3619. doi: 10.3390/nu14173619 (PMC9460668; doi:10.3390/nu14173619)
Supplement: Supplementary file 1 [file nutrients-14-03619-s001.zip › nutrients-1771677-supplementary.pdf]

Supplementary materials
Bensaada et al.

Development of an assay for Soy Isoflavones in Women's Hair

Bensaada Souad[1,2], Raymond Isabelle[1,3], Breton Malena[1], Pellegrin Isabelle[1,4], Viallard Jean-François[1,3], Bennetau-Pelissero Catherine[1,2,5]

1. University of Bordeaux, 33076 Bordeaux, France
2. ARNA, U1212 Inserm, 5320 CNRS, Pharmacy Faculty, 33076 Bordeaux, France
3. CHU Bordeaux, USN B0 - Hôpital Haut Lévêque - 33604 Pessac, France
4. CHU Bordeaux, Laboratory of Immunology and Immunogenetics, Resources Biological Center (CRB), France
5. Bordeaux Sciences Agro, 33175, Gradignan, France

Abstract: Soy isoflavones, at adequate dosage, have estrogenic and anti-thyroidal effects in animals and humans that can either be beneficial or adverse, depending on consumers' physiological status. Hence, this study present the assay of soy isoflavones in hair, aiming to give new information about a persons' isoflavones exposure, when health issues related to estrogenic or thyroidal effects are observed. Aqueous or organic extraction procedures following acidic, basic or enzymatic digestions were tested on 60 volunteer's hair samples from a hairdresser and from the clinical trial 2017T2-29. The acidic digestion method was the most efficient regarding isoflavones. A specific inquiry was developed to assess dietary habits of French consumers based on the analysis of 12,707 food labels from France. It was used to check for the reliability of the new assay method. A score for the consumer's exposure to isoflavones was built considering, among other parameters, soy-based diet and foodstuff containing soy as an ingredient, i.e. "hidden-soy". The correlation between this score and isoflavones measurements in hair reached 0.947; $p<0.001$. Therefore, providing that relevant data are considered to assess isoflavones exposure, hair which smoothen daily isoflavones intakes' variations, is a relevant tissue to assess human isoflavones exposure for subsequent health analysis.

Keywords: soy isoflavones; consumer exposure; hair samples; dietary inquiry; digestion method

Abbreviations:

DAI: Daidzein aglucone form
GEN: Genistein aglucone form
IFS: Isoflavone Food score
IHS: Isoflavone Hair score

Data presented:

Supplementary material S1: Dietary Inquiry Proposed to Volunteers

Supplementary material S2: Matrix used to Calculate IFS and IHS

Supplementary material S3: Evidencing a Questionable Answer to the Food Questionnaire

Supplementary material S4: Pearson Regression Correlation Between Individual Isoflavones and HIS

Supplementary materials
Bensaada et al.

Supplementary data S1: Dietary Inquiry

Subjet and study parameters

Study acronyme ☐ **Promotor's name** ☐

Subject code ☐ **Inquiry date** ☐

Investigator ID ☐ **Investigator's name** ☐

Hair treatments questionnaire

Sex of the volunteer ☐ **Subject's age** ☐ **Casual treatment since** ☐

Natural colour of the hair ☐ **Last colouring** ☐ **Casual product used** ☐

Shampoo frequency ☐ **Colouring frequency** ☐ **Last product used** ☐

Hair length ☐ **Last perm** ☐ **Casual product used** ☐

Cutting frequency ☐ **Perm frequency** ☐ **Last product used** ☐

Food consumption profile

Food habits

☐ Omnivorous
☐ Vegetarian
☐ Lacto-vegetarian
☐ Vegan
☐ Flexivegetarian

I eat at home
☐ 3 times a day
☐ 2 times a day
☐ once a day

I eat in a canteen
☐ 3 times a day
☐ 2 times a day
☐ once a day

I eat in a restaurant
☐ 3 times a day
☐ 2 times a day
☐ once a day

☐ I go to the fast-food once a week
☐ I eat in my office
☐ at least once a week

☐ I don't have 3 meals a day
☐ I have snacks all day
☐ I have food supplements

☐ I am a small-eater
☐ I am a medium-eater
☐ I am a big-eater

Cooking pratices	Frequency per week (number of times)
☐ Frying	☐
☐ Vapor cooking	☐
☐ Boilling	☐
☐ Grill	☐
☐ Wok/Oven	☐

Which food supplements ☐

For how-long have you been on this diet? ☐

If your case is not considered please tell us if you have a specific diet (allergies, …) ☐

Supplementary materials
Bensaada et al.

Supplementary data S1: Dietary Inquiry (continued)

Consumption of foodstuff based on "plant-milk"

1 Do you drink soy-milk?

Which season	Winter	Spring	Summer	Autumn	All year	Casual portion size

several a day	everydays	sometimes a week	once a week	< once a week	several a month	< once a month	never

Portion	Size
1/2	spoon
1	glass
2	bowl
3	plate

2 Do you eat soy-yogurts?

Which season	Winter	Spring	Summer	Autumn	All year	Casual portion size

several a day	everydays	sometimes a week	once a week	< once a week	several a month	< once a month	never

Portion	Size
1/2	spoon
1	glass
2	bowl
3	plate

3 Do you eat soy-cream?

Which season	Winter	Spring	Summer	Autumn	All year	Casual portion size

several a day	everydays	sometimes a week	once a week	< once a week	several a month	< once a month	never

Portion	Size
1/2	spoon
1	glass
2	bowl
3	plate

4 Do you eat soy-cheese or tofu?

Which season	Winter	Spring	Summer	Autumn	All year	Casual portion size

several a day	everydays	sometimes a week	once a week	< once a week	several a month	< once a month	never

Portion	Size
1/2	spoon
1	glass
2	bowl
3	plate

5 Do you drink "plant-milks" (coconut, haselnut, almond, rice...)

Which season	Winter	Spring	Summer	Autumn	All year	Casual portion size

several a day	everydays	sometimes a week	once a week	< once a week	several a month	< once a month	never

Portion	Size
1/2	spoon
1	glass
2	bowl
3	plate

Supplementary data S1: Dietary Inquiry (continued)

Consumption of elaborated dishes

6 **Do you eat soy-based products (Saussages, burgers, minced meat, balls…)**

Which season: Winter | Spring | Summer | Autumn | All year

several a day	everydays	sometimes a week	once a week	< once a week	several a month	< once a month	never

Casual portion size

1/2	spoon
1	glass
2	bowl
3	plate

7 **Do you eat meat-based elaborated dishes? (paté, sauces…)**

Which season: Winter | Spring | Summer | Autumn | All year

several a day	everydays	sometimes a week	once a week	< once a week	several a month	< once a month	never

Casual portion size

1/2	spoon
1	glass
2	bowl
3	plate

8 **Do you eat fish-based elaborated dishes? (surimi, paté...)**

Which season: Winter | Spring | Summer | Autumn | All year

several a day	everydays	sometimes a week	once a week	< once a week	several a month	< once a month	never

Casual portion size

1/2	spoon
1	glass
2	bowl
3	plate

9 **Do you eat vegetable-based elaborated dishes?**

Which season: Winter | Spring | Summer | Autumn | All year

several a day	everydays	sometimes a week	once a week	< once a week	several a month	< once a month	never

Casual portion size

1/2	spoon
1	glass
2	bowl
3	plate

10 **Do you eat pizzas?**

Which season: Winter | Spring | Summer | Autumn | All year

several a day	everydays	sometimes a week	once a week	< once a week	several a month	< once a month	never

Casual portion size

1/2	spoon
1	glass
2	bowl
3	plate

Supplementary data S1: Dietary Inquiry (continued)

11 Do you eat fresh elaborated dishes?

Which season: ☐ Winter ☐ Spring ☐ Summer ☐ Autumn ☐ All year

☐ several a day ☐ everydays ☐ sometimes a week ☐ once a week ☐ < once a week ☐ several a month ☐ < once a month ☐ never

Casual portion size: ☐ 1/2 ☐ 1 ☐ 2 ☐ 3 — ☐ spoon ☐ glass ☐ bowl ☐ plate

12 Do you eat meat-based canned dishes?

Which season: ☐ Winter ☐ Spring ☐ Summer ☐ Autumn ☐ All year

☐ several a day ☐ everydays ☐ sometimes a week ☐ once a week ☐ < once a week ☐ several a month ☐ < once a month ☐ never

Casual portion size: ☐ 1/2 ☐ 1 ☐ 2 ☐ 3 — ☐ spoon ☐ glass ☐ bowl ☐ plate

13 Do you eat nuggets frozen or not?

Which season: ☐ Winter ☐ Spring ☐ Summer ☐ Autumn ☐ All year

☐ several a day ☐ everydays ☐ sometimes a week ☐ once a week ☐ < once a week ☐ several a month ☐ < once a month ☐ never

Casual portion size: ☐ 1/2 ☐ 1 ☐ 2 ☐ 3 — ☐ spoon ☐ glass ☐ bowl ☐ plate

14 Do you eat Kebbabs or hamburgers?

Which season: ☐ Winter ☐ Spring ☐ Summer ☐ Autumn ☐ All year

☐ several a day ☐ everydays ☐ sometimes a week ☐ once a week ☐ < once a week ☐ several a month ☐ < once a month ☐ never

Casual portion size: ☐ 1/2 ☐ 1 ☐ 2 ☐ 3 — ☐ spoon ☐ glass ☐ bowl ☐ plate

15 Do you eat meat-balls frozen or not?

Which season: ☐ Winter ☐ Spring ☐ Summer ☐ Autumn ☐ All year

☐ several a day ☐ everydays ☐ sometimes a week ☐ once a week ☐ < once a week ☐ several a month ☐ < once a month ☐ never

Casual portion size: ☐ 1/2 ☐ 1 ☐ 2 ☐ 3 — ☐ spoon ☐ glass ☐ bowl ☐ plate

Supplementary data S1: Dietary Inquiry (continued)

16 Do you eat "escaloppe cordon bleu" frozen or not?

Which season ☐ **Winter** ☐ **Spring** ☐ **Summer** ☐ **Autumn** ☐ **All year**

☐ several a day ☐ everydays ☐ sometimes a week ☐ once a week ☐ < once a week ☐ several a month ☐ < once a month ☐ never

Casual portion size

☐ 1/2 ☐ 1 ☐ 2 ☐ 3

☐ spoon ☐ glass ☐ bowl ☐ plate

17 Do you eat stuffed vegetables frozen or not?

Which season ☐ **Winter** ☐ **Spring** ☐ **Summer** ☐ **Autumn** ☐ **All year**

☐ several a day ☐ everydays ☐ sometimes a week ☐ once a week ☐ < once a week ☐ several a month ☐ < once a month ☐ never

Casual portion size

☐ 1/2 ☐ 1 ☐ 2 ☐ 3

☐ spoon ☐ glass ☐ bowl ☐ plate

18 Do you eat frozen ground beef ?

Which season ☐ **Winter** ☐ **Spring** ☐ **Summer** ☐ **Autumn** ☐ **All year**

☐ several a day ☐ everydays ☐ sometimes a week ☐ once a week ☐ < once a week ☐ several a month ☐ < once a month ☐ never

Casual portion size

☐ 1/2 ☐ 1 ☐ 2 ☐ 3

☐ spoon ☐ glass ☐ bowl ☐ plate

19 Do you eat industrial biscuits?

Which season ☐ **Winter** ☐ **Spring** ☐ **Summer** ☐ **Autumn** ☐ **All year**

☐ several a day ☐ everydays ☐ sometimes a week ☐ once a week ☐ < once a week ☐ several a month ☐ < once a month ☐ never

Casual portion size

☐ 1/2 ☐ 1 ☐ 2 ☐ 3

☐ spoon ☐ glass ☐ bowl ☐ plate

Supplementary data S1: Dietary Inquiry (continued)

Legume consumption raw or cooked

20 Do you eat legumes like beans, lentils, peas

Which season: Winter / Spring / Summer / Autumn / All year

several a day / everydays / sometimes a week / once a week / < once a week / several a month / < once a month / never

Casual portion size: 1/2 / 1 / 2 / 3 — spoon / glass / bowl / plate

21 Do you eat chick-peas?

Which season: Winter / Spring / Summer / Autumn / All year

several a day / everydays / sometimes a week / once a week / < once a week / several a month / < once a month / never

Casual portion size: 1/2 / 1 / 2 / 3 — spoon / glass / bowl / plate

22 Do you eat soybeans ?

Which season: Winter / Spring / Summer / Autumn / All year

several a day / everydays / sometimes a week / once a week / < once a week / several a month / < once a month / never

Casual portion size: 1/2 / 1 / 2 / 3 — spoon / glass / bowl / plate

23 Do you eat mungo-sprouts?

Which season: Winter / Spring / Summer / Autumn / All year

several a day / everydays / sometimes a week / once a week / < once a week / several a month / < once a month / never

Casual portion size: 1/2 / 1 / 2 / 3 — spoon / glass / bowl / plate

24 Do you eat alfalfa or clover sprouts?

Which season: Winter / Spring / Summer / Autumn / All year

several a day / everydays / sometimes a week / once a week / < once a week / several a month / < once a month / never

Casual portion size: 1/2 / 1 / 2 / 3 — spoon / glass / bowl / plate

Supplementary material S2: Calculation of IFS and HIS

Hair

						Multiplying factor
Length	Short 0,5	Medium 0,2	Long 0,1		A	a = A + B + C
Frequency of cutting	<2 month 0,5	<4 month 0,2	>4 month 0,1		B	
Treatments	Shampoo 0,5	Colouring 0,2	Perm 0,1		C	

Dietary tendencies

						Multiplying factor
Big eater 1,5	Medium eater 1	Small eater 0,5			D	b = D

c = a + b

Diet duration (months) E		Hair length F			G=E/F

Vegan 1	Vegetarian 0,8	Lactovegetarian 0,8	Flexitarian 0,5	Omnivore 0,2	a
Asian 1	Exotic 0,8	Gluten free 0,2	Lactose free 0	Other	b
Wok/Oven 1	Vapor 0,8	Grill 0,5	Frying 0,2	Boiling 0	c
Ultratransformed 1	Industrial 0,5	Home-made 0,2			d
Canteen >3 times/week 1	Fast food >2times /w 0,8	Restaurant > 2times/w 0,5	Home > 3times/w 0,2		e

Supplementary material S2: Calculation of IFS and HIS (continue)

Soy-consumption

Soy juice	Every day 2	> once a week 1	< once a week 0,5	never 0	f	
Soy yogurt	Every day 0,8	> once a week 0,3	< once a week 0,1	never 0	g	
Soy cream and icecream	Every day 2	> once a week 1	< once a week 0,5	never 0	h	
Soy cheese and tofu	Every day 1	> once a week 0,5	< once a week 0,2	never 0	i	
Other plant juices	Every day 0	> once a week 0	< once a week 0	never 0	j	
Other soy products (miso, tempeh…)	Every day 2	> once a week 1	< once a week 0,5	never 0	k	
Meat based dishes	Every day 0,2	> once a week 0,1	< once a week 0	never 0	l	If Canteens, increased to the upper level
Fish based dishes	Every day 0,2	> once a week 0	< once a week 0	never 0	m	If Canteens, increased to the upper level
Vegetables based dishes	Every day 0,4	> once a week 0,2	< once a week 0	never 0	n	If Canteens, increased to the upper level
Pizzas	Every day 0,1	> once a week 0	< once a week 0	never 0	o	If Canteens, increased to the upper level
Elaborated dishes	Every day 0,3	> once a week 0,1	< once a week 0	never 0	p	If Canteens, increased to the upper level
Canned recipes	Every day 0,2	> once a week 0,1	< once a week 0	never 0	q	If Canteens, increased to the upper level
Nuggets	Every day 0,2	> once a week 0,1	< once a week 0	never 0	r	If soy increased to the upper level
Hamburgers / Kebabs	Every day 0,5	> once a week 0,2	< once a week 0,1	never 0	s	If soy increased to the upper level
Meat balls	Every day 0,5	> once a week 0,2	< once a week 0,1	never 0	t	If soy increased to the upper level

Supplementary materials
Bensaada et al.

Supplementary material S2: Calculation of IFS and HIS (continue)

Escalope cordon bleu	Every day 0,2	> once a week 0,1	< once a week 0	never 0	u	If soy increased to the upper level
Stuffed vegetables (tomatoes, cabbages…)	Every day 0,2	> once a week 0,1	< once a week 0	never 0	v	If Canteens, increased to the upper level
Frozen minced steaks	Every day 0,5	> once a week 0,2	< once a week 0,1	never 0	w	If Canteens, increased to the upper level
Industrial biscuits	Every day 0,15	> once a week 0,1	< once a week 0	never 0	x	If Canteens, increased to the upper level
Other legumes	Every day 0,2	> once a week 0,1	< once a week 0	never 0	y	
Chick peas	Every day 0,2	> once a week 0,1	< once a week 0	never 0	z	
Soy seeds	Every day 3	> once a week 2	< once a week 1	never 0	aa	
soybean sprouts	Every day 0,5	> once a week 0,2	< once a week 0,1	never 0	ba	
Alfalfa or clover sprouts	Every day 3	> once a week 2	< once a week 1	never 0	ca	

Food supplements	Soy/Alfalfa/Clover 1	Others 0	None 0		da

Isoflavone Food Score (IFS) **IFS=SUM(a:da)* c**

Isoflavone Hair Score (IHS) **IHS=IFS*G**

Supplementary materials
Bensaada et al.

Supplementary material S3: Evidencing a Questionable Answer to the Food Questionnaire

A

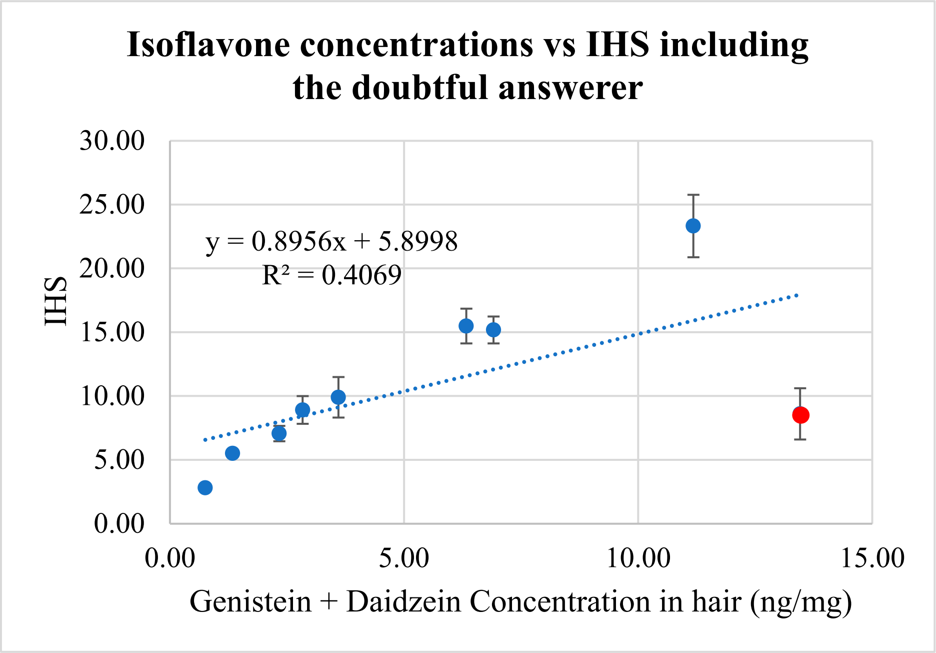

N= 9; r = 0.638 p<0.1

B

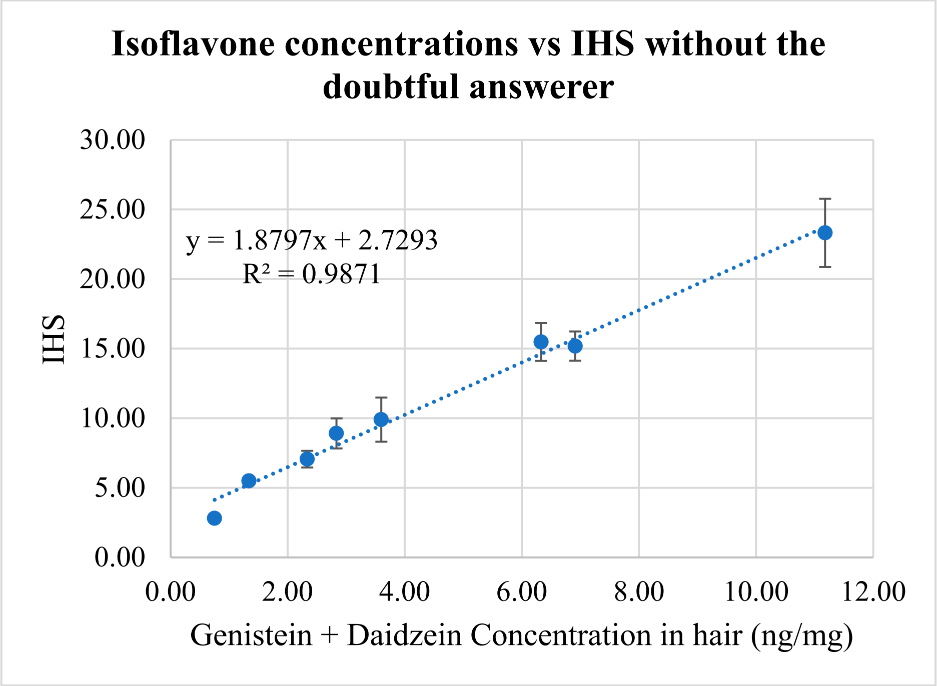

N = 8; r = 0.993 p<0.001

Supplementary materials
Bensaada et al.

Supplementary material S4: Pearson Regression Correlation Between Individual Isoflavones and IHS

A

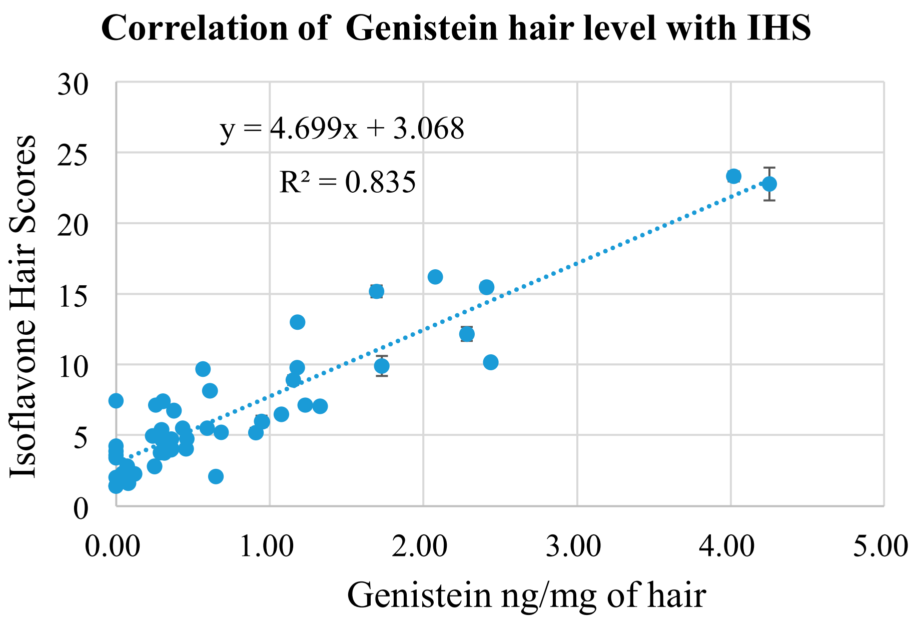

N = 60; $r_G = 0.914$ $p < 0.001$

B

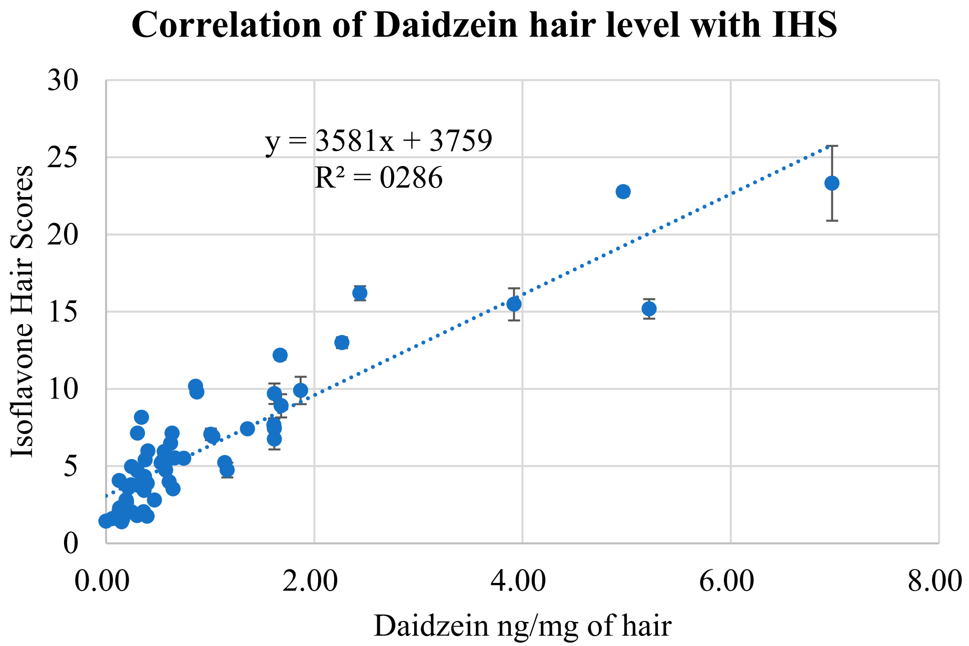

N = 60; $r_D = 0.91$; $p < 0.001$
